# The Years That Count

**Published:** 1923-07

**Authors:** George H. Green

**Affiliations:** Lecturer in Education, University College of Wales


### THE YEARS THAT COUNT.

**By GEORGE H. GREEN, B.Sc., B.Litt., Lecturer in Education in the University College of Wales.**

RATHER more than a century ago Captain Cook knew that if he could secure at reasonable intervals a diet of fresh meat and vegetables for his men he could make voyages of very great length without risk of those outbreaks of scurvy which the majority of navigators of that day regarded as inevitable. Later, the lime-juice ration was found to have the same effect. Nowadays we are beginning to know something of the reason. There are still people who ask patronisingly, in connection with the discoveries of modern science, how it is that people have so lately began to grow wise. This is particularly the case in connection with the claim that only now are we beginning to understand the mind of the child. Newspapers still point out that people who have babies must know all about them. Yet, if this were true, the medical profession would not have had its long and uphill fight in the cause of infant welfare and against high infant mortality. Nor would there be any need of a Baby Week—this year from July 1 to July 7—to popularise the modern knowledge concerning the needs of the infant.

### A Child's Mental Needs.

Precisely parallel with the popular ignorance about the physical needs of the child has run the popular ignorance about its mental needs. Those who have had the care of children have perhaps known less about the workings of its mind than those of its body. As regards the training of children, it was more difficult to arrive at a science than in the case of their bodies. It is very difficult to discover what factor in a man's history was the cause of something in his character that we are attempting to study. At sixty, for example, one man is a saint and another a satyr. What part in either of these final results is due to heredity and what part to training ? And of this latter, which part is due to home influences, which to school, which to companions, which to the University years, which to early experiences in business or profession, which to material circumstances ?

### The Early Years.

The observations of those who were responsible for the training of children made it clear that the character was influenced by the events and the training of earlier years. "As the twig is bent the tree's inclined" expressed a widely shared belief. But what period was the formative one, that on which all later development depended ? The men who claimed that if they were given the child for the first twelve years of its life they cared little enough who carried on the training they had begun were generally regarded as guilty of over-statement. The emphasis that certain writers tended to place on the years of adolescence led a great many people to believe that these trying years were the all-important ones. But in the years preceding the war a new method of treating the psycho-neuroses had developed in the hands of a small group of physicians, and one of these, Freud, had endeavoured to state a theory as to the nature and origin of these diseases. The theory itself does not matter greatly here. There are still many differences of opinion about parts of it. The statements of Freud, Jung, Adler and Rivers are by no means the same. But there is one point about which all are agreed, however they may differ in other respects—the early years of a child's life are critical for its later development. In the past it is these years that have been regarded as of least importance. Looking after the child has meant merely keeping him quiet and out of mischief.

### The Fight of the Future.

The psychological importance of these years is not yet widely understood. The vicious idea that anyone can look after a young child is the result of the obsession with the Gradgrind view that education is nothing more than a matter of "learning lessons." Men do not realise that it is of far more importance to surround the very young child with an environment in which it may be active, in which its mind and body may develop, in which the formation of its character may begin, than it is for it to "learn" things that can be acquired easily and quickly later on. An essential part of such an environment is a skilled and understanding directress, whose selection and training are of immense importance. Baby Week represents an annual effort to popularise this view, to present it in a form that is suited to the understanding of those who are not specialists in these fields, but who, as the mothers and fathers of the children, are immediately concerned.

---

### NEW LABORATORIES AT ST. THOMAS'S HOSPITAL.

IN the absence of the Duke of Connaught, President of the Hospital, the Marquess of Cambridge presented the prizes to the students of St. Thomas's Medical School on Wednesday, July 13. The occasion was one of special importance, as it marked the public acceptance of the new laboratory, the gift of the Dunn Trustees. Lord Cambridge having inspected the laboratory, Sir Walter Fletcher gave an account of the work that would be done there. The apparatus of a well-equipped chemical laboratory was, he said, becoming as essential to the daily work of the physician as the stethoscope in his pocket, or his microscope and the aids of bacteriology. Sir Jeremiah Colman explained that Sir William Dunn was a very successful South African merchant, who appointed the directors of the Commercial Union Insurance Co. to decide how his fortune should be distributed. On behalf of the Trustees, Sir Jeremiah asked acceptance of the laboratory, and, amid great applause, he offered a further £5,000 for scientific instruments. The Chairman, Sir Arthur Stanley, having accepted the gifts, the Marquess of Cambridge distributed the prizes and the guests left the Governors' Hall for the terrace, where tea was served and the band of the Grenadier Guards played.

